# Feature-preserving simplification framework for 3D point cloud

**DOI:** 10.1038/s41598-022-13550-1

**Published:** 2022-06-08

**Authors:** Xueli Xu, Kang Li, Yifei Ma, Guohua Geng, Jingyu Wang, Mingquan Zhou, Xin Cao

**Affiliations:** 1 School of Information Science and Technology, Northwest University, Xi’an, 710127 Shaanxi China; 2 Yan’an University, Yan’an, 716000 Shaanxi China; 3 National and Local Joint Engineering Research Center for Cultural Heritage Digitization, Xi’an, 710127 Shaanxi China

**Keywords:** Applied optics, Mathematics and computing

## Abstract

To obtain a higher simplification rate while retaining geometric features, a simplification framework for the point cloud is proposed. Firstly, multi-angle images of the original point cloud are obtained with a virtual camera. Then, feature lines of each image are extracted by deep neural network. Furthermore, according to the proposed mapping relationship between the acquired 2D feature lines and original point cloud, feature points of the point cloud are extracted automatically. Finally, the simplified point cloud is obtained by fusing feature points and simplified non-feature points. The proposed simplification method is applied to four data sets and compared with the other six algorithms. The experimental results demonstrate that our proposed simplification method has the superiority in terms of both retaining geometric features and high simplification rate.

## Introduction

With the improvement of 3D data acquisition capabilities^1–3^, point cloud models are gaining increasing attention and being widely utilized in many fields, such as scene reconstruction^4–9^, museum display^10–13^, and virtual projection^14–18^. High precision of the 3D scanning technology can provide abundant details of the point clouds, but it will simultaneously generate a mass of redundant data, which seriously reduces the efficiency of data processing, transmission and display, thereby affecting subsequent applications.

The previous research on point cloud simplification methods can be generally classified into two kinds. One is mesh-based and the other is scattered-point-based. The mesh-based methods convert the point cloud to the mesh model with polygons, and then reduce the points based on specific rules for simplification. Hamann^19^ developed an algorithm to iteratively delete triangles according to the triangulation, and it had a good effect on the model surface. Lounsbery et al.^20^ simplified the connected triangular mesh through wavelet representation. Weir et al.^21^ proposed a simplification algorithm based on the bounding box. The algorithm constructed a bounding box that surrounded all the points of the 3D model, and the bounding box was divided into several small cubes evenly, and the closest point from the center of the small cube replaced all other points. The implement is simple and easy, however, different point clouds need different division scales, and the simplification accuracy cannot be guaranteed. Kalvin and Taylor^22^ proposed a bounded approximation method, which placed the vertices of the polyhedral mesh in an error region, and this method improved the practical feasibility. Gong et al.^23^ combined voxel grid with the bounding box, and confirmed the center of each small grid by calculating the distance of k neighborhood and the normal for simplification. This method is apt to discard feature points in the area where the curvature changes. The main disadvantages of these mesh-based methods are that they are seriously complex and building polygonal structural meshes require a great quantity of extra information and memory space. In contrast, the scattered-point-based methods can consume the point cloud directly. These methods can mainly be summarized into two categories. One is based on the global ideology for simplification, and the other is based on the partition strategy, extracting the feature points before simplification. Song and Feng^24^ reduced the points globally according to the specified simplification ratio. Shi et al.^25^ proposed a simplification method based on k-means clustering. Xiao and Huang^26^ proposed a kd-tree-based method that uniformly simplified the point cloud. Although the simplification efficiency is high, the threshold needs to be adjusted according to the specific model. Zin et al.^27^ presented a simplification method based on the unit normal vector. The feature points were extracted by constructing boundary spheres to search for k nearest neighbors and measuring the curvature of each point. Wei^28^ established the tangent plane based on the least squares fitting method^29^, analyzed the geometric distribution characteristics of the points on the projection surface according to the relationship between the points, and detected the edge feature points. Zanger et al.^30^ presented a multi-level method for preserving geometric features of different scales. Han et al.^31^ proposed an edge-preserving algorithm. Elkhrachy^32^ segmented the edges of the point cloud by the normal vector. This method detected the adjacent normals according to the threshold to determine the edge points. Chen and Sun^33^ proposed a method that divided the original point cloud into spaces, constructed the k neighborhood of the point, set parameters of features for analysis, and combined the local average distance with the contours of the edge points for classification. The prime shortage of the scattered-point-based methods is that they neglect the intrinsic correlation between points of the point cloud owing to their lack of topological structures, resulting in the loss of some significant geometric characteristics.

**Figure 1:**
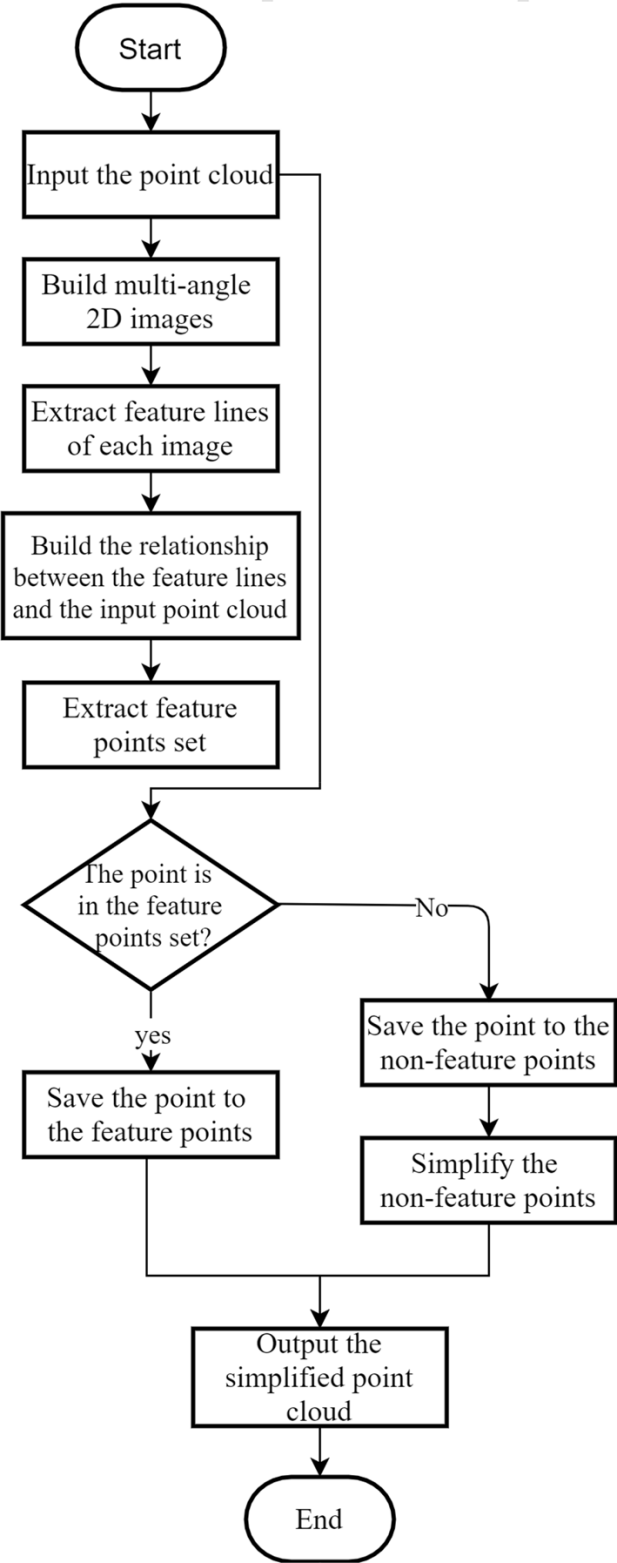
Flowchart of the proposed simplification framework.

Here, a novel simplification framework for the point cloud is presented. It consists of two parts, the feature points and the simplified non-feature points. Feature points, which are vital for representing the geometric features of the point cloud, are extracted through three steps. The first is obtaining multi-angle images of the point cloud; the second is extracting feature lines of each image; the third is obtaining the feature points from the original point cloud based on the extracted feature lines. The simplified non-feature points, which are utilized for filling the flat areas of the 3D model to maintain its integrity, are extracted from the subset of the point cloud except for feature points. The flowchart of the proposed framework is shown in Fig. 1.

Experimental results demonstrate that the proposed framework can achieve a simplified point cloud with high quality. The main contributions of this work can be summarized as:

1. A feature-line based framework is proposed for point cloud simplification. Inspired by the success of deep learning in extracting critical image features, a method that transforms 3D point cloud to 2D images is proposed to better learn the characteristics of the point cloud.
2. The mapping relationship between the images and the point cloud is presented. According to the correspondence between the point cloud and images, one pixel in the image is related to a group of 3D points (one-to-k), and a novel method is proposed to receive the final correspondence (one-to-one).
3. Large numbers of experiments demonstrate that the framework proposed in this paper has obvious advantages and achieves higher simplification rate and better simplification effect simultaneously.

## Experiments and results

### Experiments settings

To evaluate the performance of the proposed framework, the simplified results of our method are compared with the results of the other six simplified methods: DFPSA^34^, Gaussian spheres^35^, octree coding^36^, k-means clustering^37^, uniform simplification^38^, and geometric algebra^39^. The experiment uses two platforms. One is windows10: CPU is Intel(R) Xeon(R) E5-2650 v3 @2.30 GHz, 32 GB memory, mainly for running Matlab code and processing related model software; the other is Ubuntu 18.04: CPU is Intel(R) Core i7-9700 @3.00 GHz, 64 GB memory, graphics card is NVIDIA GeForce RTX 2080Ti, mainly used for debugging and running related deep learning code with python.

### The choice of the axis and angle for capturing 2D images

In order to analyze the influence of the coordinate axis, the experiment fixes the rotation angle at 60° to extract the feature points of the X-axis, X/Y-axis, and X/Y/Z-axis respectively. The results of different feature points are shown in Table 1:

**Table 1:** Results of different feature points under X-axis, X/Y-axis, and X/Y/Z-axis with 60°.

	X-axis	X/Y-axis	X/Y/Z-axis	Original model
Bunny	1657	3126	3982	35,944
Elephant	1162	2423	3347	24,950
Gargo50k	958	1580	2166	25,036
Horse	1883	3401	4324	48,447

**Figure 2:**
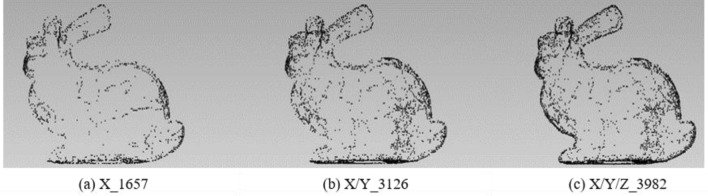
The point cloud with different numbers of feature points extracted with different axes for Bunny.

**Figure 3:**
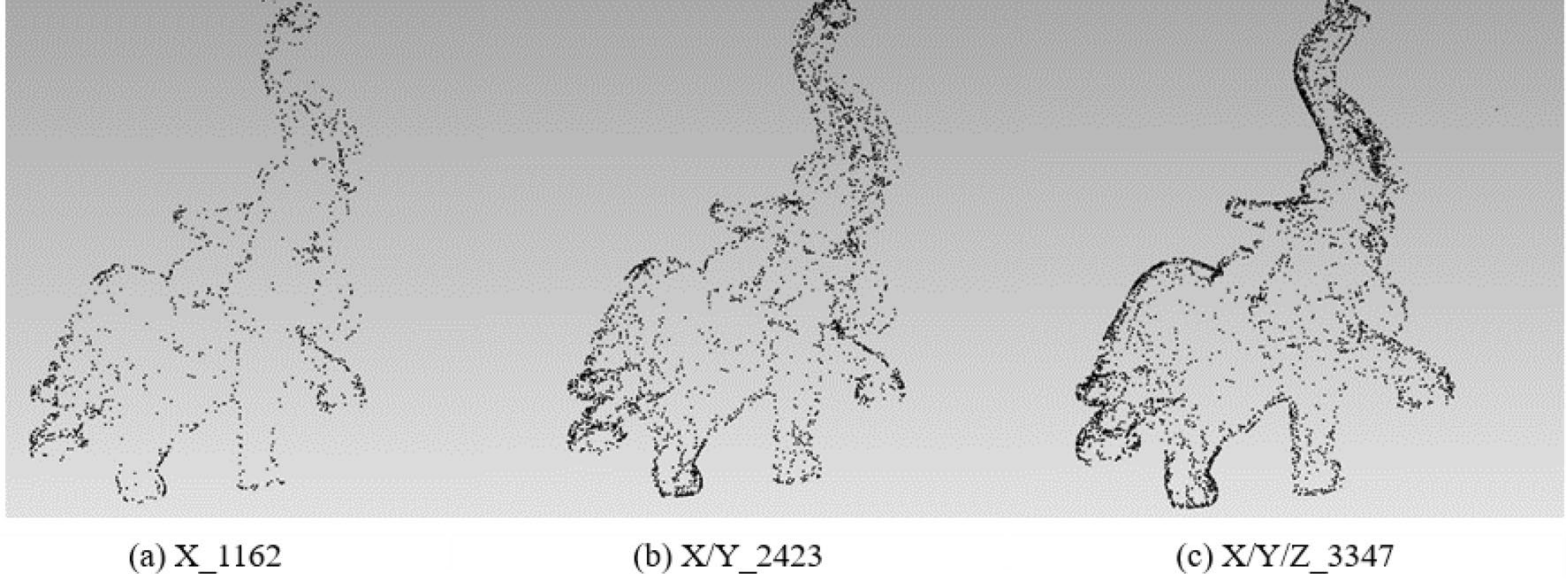
The point cloud with different numbers of feature points extracted with different axes for Elephant.

**Figure 4:**
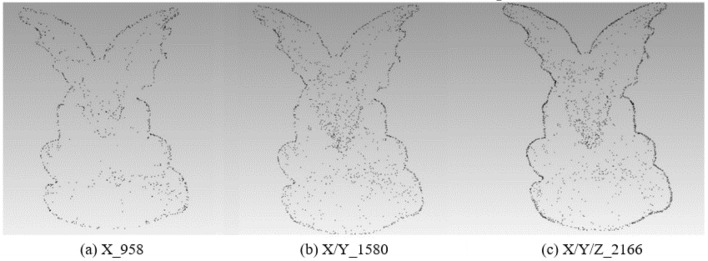
The point cloud with different numbers of feature points extracted with different axes for Gargo50k.

**Figure 5:**
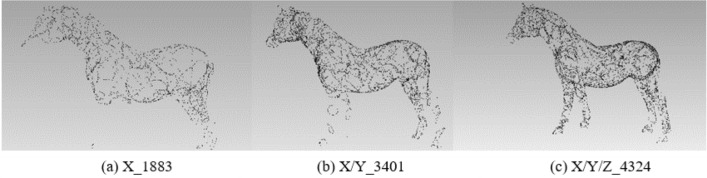
The point cloud with different numbers of feature points extracted with different axes for Horse.

Figures 2, 3, 4 and 5 illustrates the point cloud with different numbers of the feature points extracted with different axes for Bunny, Elephant, Gargo50k and Horse. For example, in Fig. 2, X_1657 represents that the original point cloud model is rotated around the X-axis to capture the corresponding 2D images, and 1657 is the feature points extracted from the original point cloud based on the mapping relationship between 2D images to the 3D model. X/Y_3126 shows that the 3D model is rotated around the X-axis and Y-axis to capture the 2D images, and the number 3126 is similar to the number in X_1657. The meaning of X/Y/Z_3982 is similar. The labels in other pictures are with same meaning.

From Table 1 and Figs. 2, 3, 4 and 5, it can be known that with the increase of information provided by the X-axis, X/Y-axis, and X/Y/Z-axis, the number of feature points increases significantly, and the corresponding information increments provided by the X-axis and X/Y-axis are obviously more than that of the X/Y/Z-axis. It is obvious that Stanford’s model is regular based on the X/Y/Z-axis. The information of most points is concentrated on the X-axis and Y-axis, and the information on the Z-axis is naturally relatively small. However, as most of the models do not satisfy the standard X/Y-axis coordinates, the information on the Z-axis is also very important, and the Z-axis information should be retained. Moreover, under the unified mode of X/Y/Z-axis, not only the integrity of the 3D point cloud feature information is guaranteed, but also the irregular model does not need to be initialized, eliminating some troublesome preprocessing processes.

To analyze the impact of different rotation angles, the experiment fixed the rotation axis with the X/Y/Z-axis to extract the feature points of the angles 90°, 60°, 45°and 30° respectively. The results of different feature points are shown in Table 2:

**Table 2:** Results of different feature points under rotation angles 90°, 60°s, 45° and 30° with X/Y/Z-axis.

	90°	60°	45°	30°	Original model
Bunny	2123	3982	4658	6641	35,944
Elephant	1870	3347	4554	6285	24,950
Gargo50k	1505	2166	2719	3809	25,036
Horse	4620	6726	9402	13,211	48,447

**Figure 6:**
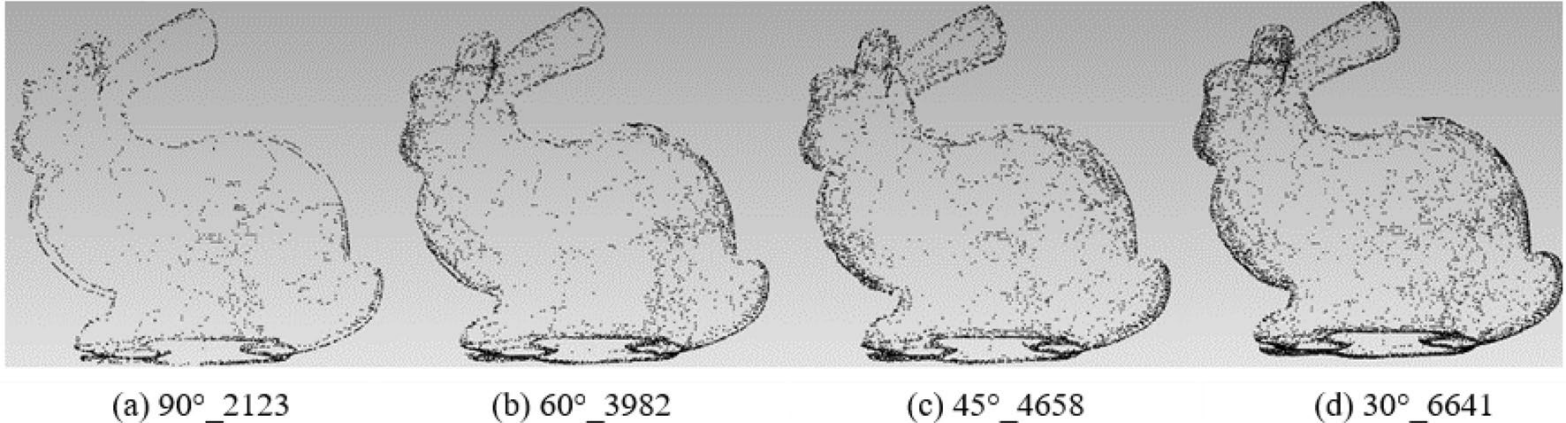
The point cloud with different numbers of feature points extracted with different angles for Bunny.

**Figure 7:**
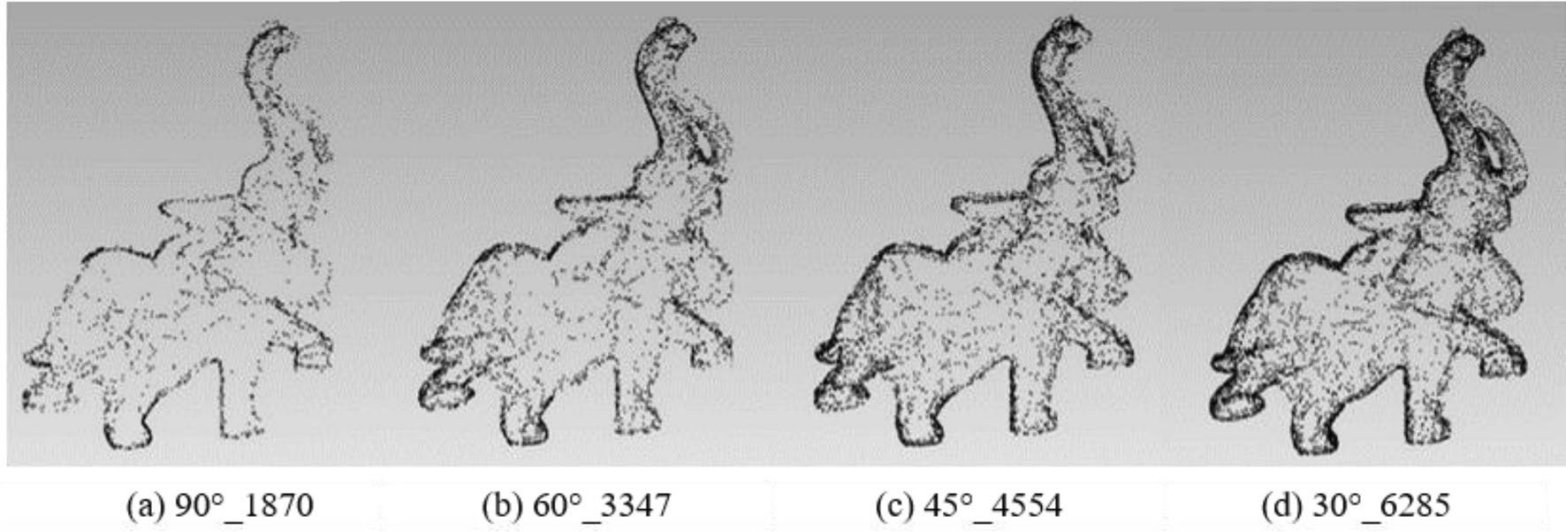
The point cloud with different numbers of feature points extracted with angles for Elephant.

**Figure 8:**
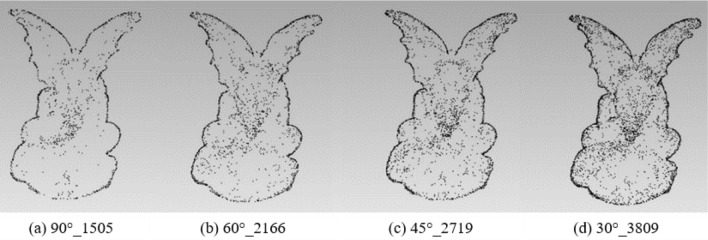
The point cloud with different numbers of feature points extracted with different angles for Gargo50k.

**Figure 9:**
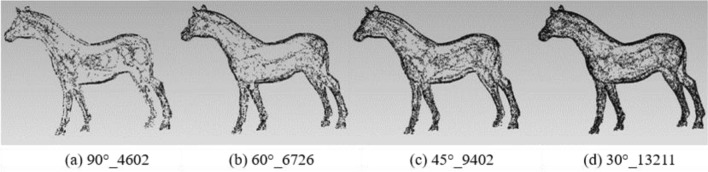
The point cloud with different numbers of feature points extracted with different angles for Horse.

Figures 6, 7, 8 and 9 illustrates the point cloud with different numbers of the feature points extracted with different rotation angles for Bunny, Elephant, Gargo50k and Horse. For example, in Fig. 6, 90°_2123 represents that the original point cloud model is rotated with angle 90° to capture the corresponding 2D images, and 2123 is the feature points extracted from the original point cloud based on the mapping relationship between the images to the point cloud. The meanings of other labels in figures are similar.

Table 2 shows that as the angle gradually increases, the information of the corresponding captured image is less, and the feature points extracted from the original point cloud are also fewer. Therefore, the smaller the angle is, the more the feature points are, and the higher the fineness of the image would be. However, with the number of images increasing, the information redundancy gets worse, resulting in time-consuming and complex calculation to achieve the goals. Therefore, it is essential to balance the rotation angles and the number of images.

From the Figs. 6, 7, 8 and 9, it can be found that the result with rotation angle 60° not only satisfies the geometric characteristics retaining of the point cloud compared to the results of 90°, but also reduces time consumption compared to the results of 45° and 30°. Therefore, X/Y/Z-axis and 60° are to be the best choice of the axis and angle for capturing 2D images.

### The choice of parameters for extracting feature points of the 3D point cloud

Based on the predicted feature images, there are three parameters $\alpha$, $\beta$ and $\gamma$ together constraining the feature point extraction effect. $\alpha$ controls the threshold of the predicted grayscale feature image to decide whether each pixel belongs to the feature line. $\beta$ controls the width of the feature line. $\gamma$ controls the spatial size of each feature pixel in the 2D image corresponding to the 3D model. The influence of different parameters for extracting feature points of the 3D point cloud (bunny for instance) is shown in Table 3:

**Table 3:** The number of feature points with different parameters α, β, γ.

α	β	γ	Feature points	α	β	γ	Feature points	α	β	γ	Feature points	Approximation
0.2	2	0.5	6925	0.23	2	0.5	7097	0.25	2	0.5	7270	7000
0.2	2	0.33	3885	0.23	2	0.33	3982	0.25	2	0.33	4088	4000
0.2	2	0.25	2408	0.23	2	0.25	2471	0.25	2	0.25	2531	2500
0.2	2	0.2	1659	0.23	2	0.2	1705	0.25	2	0.2	1748	1700
0.2	3	0.5	2859	0.23	3	0.5	3064	0.25	3	0.5	3289	3000
0.2	3	0.33	1462	0.23	3	0.33	1565	0.25	3	0.33	1687	1700
0.2	3	0.25	882	0.23	3	0.25	944	0.25	3	0.25	1007	1000
0.2	3	0.2	593	0.23	3	0.2	635	0.25	3	0.2	680	600

**Figure 10:**
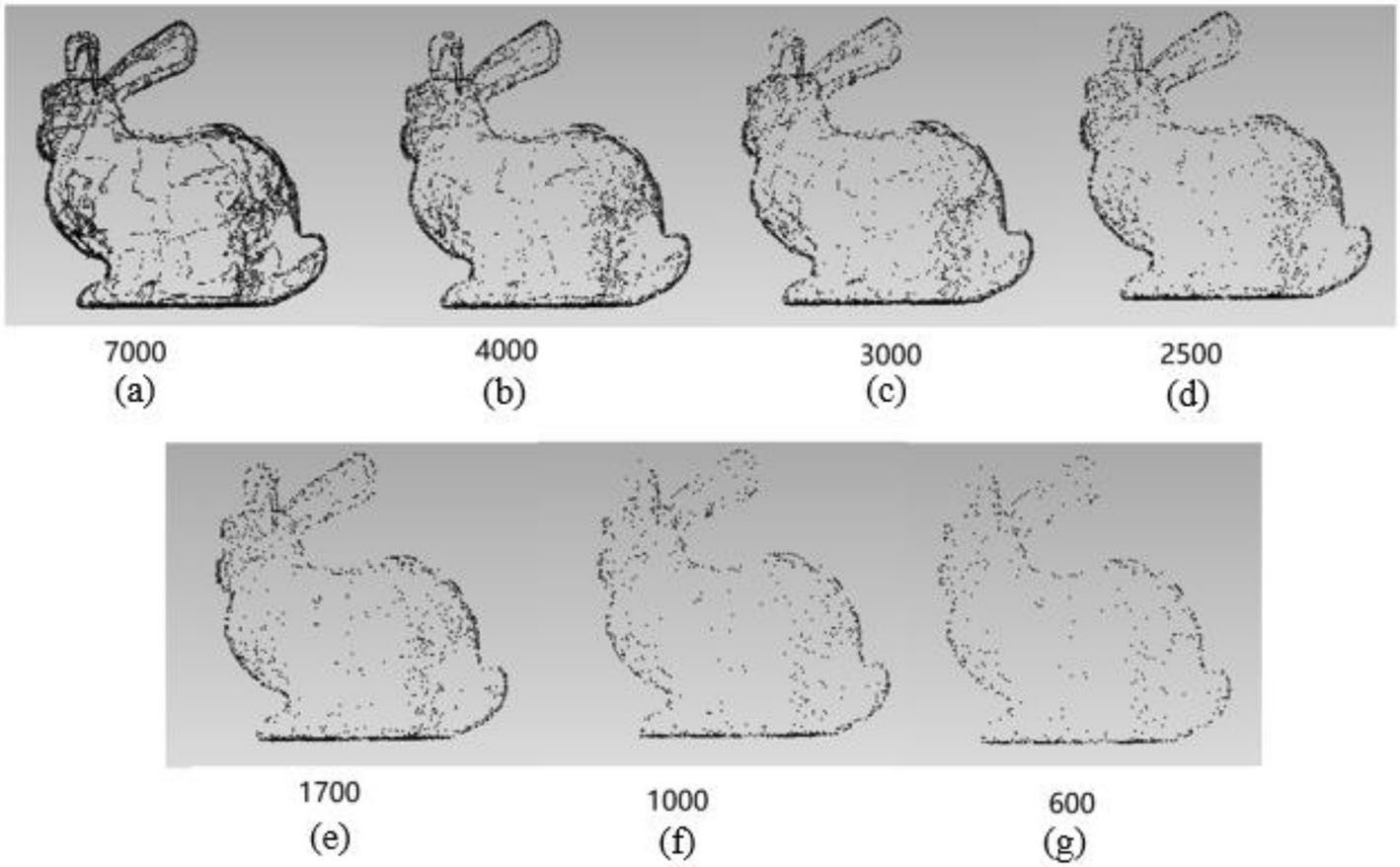
The feature extraction results with different parameters.

Table 3 shows that different parameters have different effects on extracting the feature points. The change of $\alpha$ has little effect on the number of feature points, while $\beta$ and $\gamma$ bring great difference. Figure 10 shows the feature extraction results of bunny with approximate feature points.

**Figure 11:**
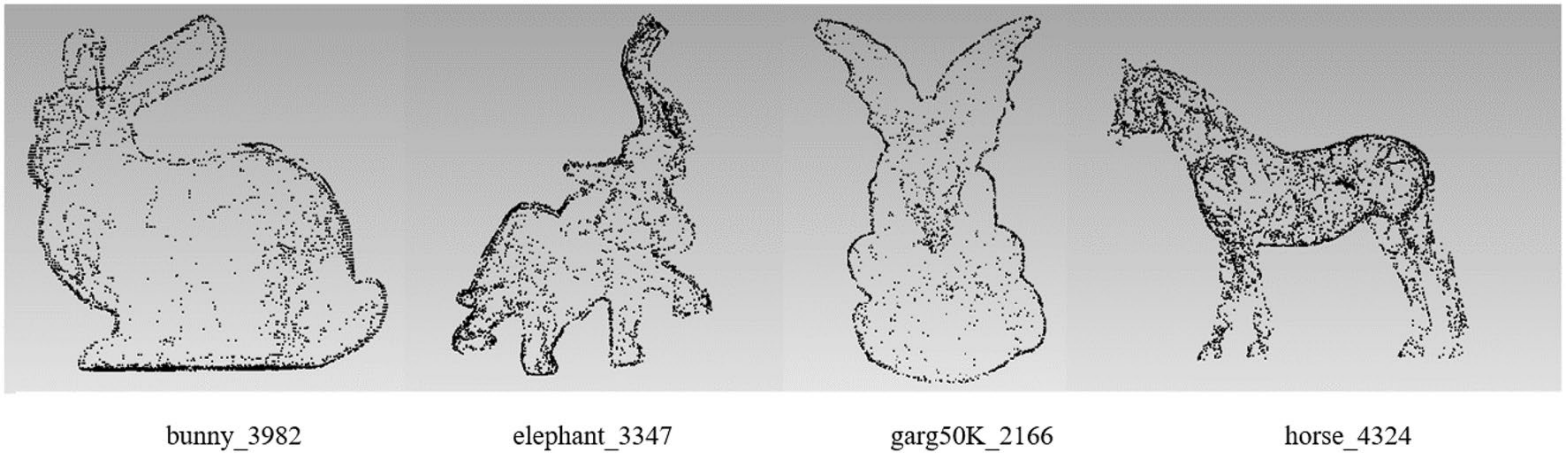
Feature points extraction results. The number of feature points of bunny = 3982, elephant = 3347, garg50K = 2166, and horse = 4324.

It can be seen that the point clouds are too sparse in Fig. 10e–g, and the contours are not complete enough to cover all feature points. However, (a) has too many points and does not meet the requirements of simplification. The ears in (c) and (d) are not perfect, and there are holes in both. In contrast, (b) shows best among them, not only retaining the contour points, but also showing more details such as ears, neck and bottom. Therefore, we select the parameters with the number of feature points around 4000. There are three groups of such parameters in Table 3, $\alpha = 0.2,\beta = 2,\gamma = 0.33$; $\alpha = 0.23,\beta = 2,\gamma = 0.33$ and $\alpha = 0.25,\beta = 2,\gamma = 0.33$. A series of experiments on other models with these parameters are carried out and it indicates that the results are most stable with parameters $\alpha = 0.23,\beta = 2,\;{\rm and}\;\gamma = 0.33$. Therefore, we adopt $\alpha = 0.23,\beta = 2,\;{\rm and}\;\gamma = 0.33$ as the optimal ones. The feature point extraction results obtained with these optimal parameters are shown in Fig. 11.

## Results

To show the simplification results more intuitively, the simplified point cloud is reconstructed to the 3D model. To further verify the superiority, our method is compared with other existed ones.

**Figure 12:**
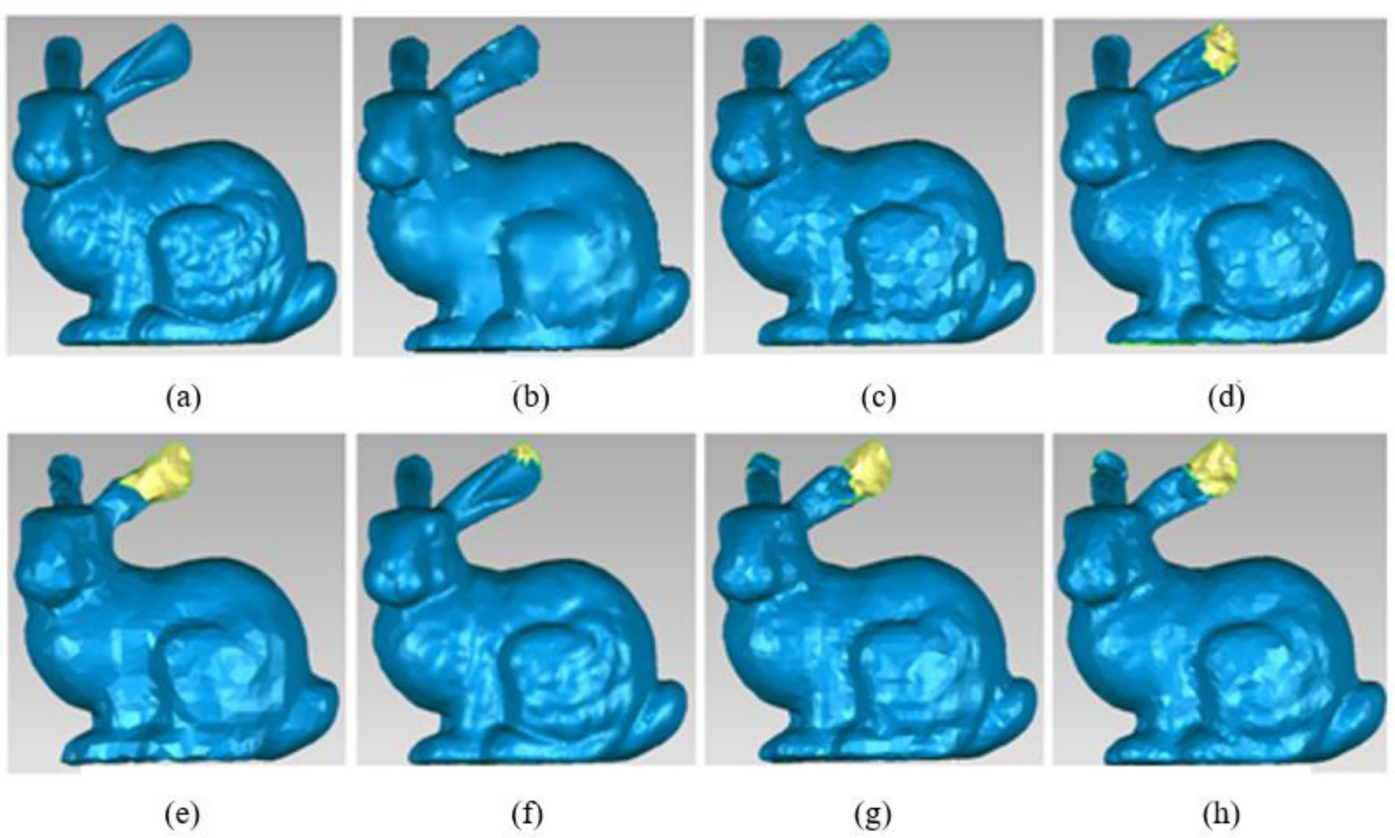
Different simplified results of Bubby. (**a**) Original data. (**b**) Our method. (**c**) The simplified method based on DFPSA. (**d**) The simplified method based on Gaussian spheres. (**e**) The simplified method based on octree coding. (**f**) The k-means clustering simplification method. (**g**) The uniform simplification method. (**h**) The geometric algebra method.

Figure 12 shows the reconstruction results of the simplified bunny. (a) Original data, total number of the points = 35,947, (b) Our method, total number of the points = 7000, (c) The simplified method based on DFPSA, total number of the points = 6730, (d) The simplified method based on Gaussian spheres, total number of the points = 8491, (e) The simplified method based on octree coding, total number of the points = 3005, (f) The k-means clustering simplification method, total number of the points = 17,385, (g) The uniform simplification method, total number of the points = 4539, (h) The geometric algebra method, total number of the points = 5434.

Figure 12 reveals that the simplification methods based on Gaussian spheres, octree coding, k-means, uniform simplification, and geometric algebra result in serious holes at the end of bunny’s ears. For DFPSA, a pair of ears looks complete and has the same outline as the original model, but there are still some holes on the corners. Our method looks smoother and the total number of simplified points is similar with that of DFPSA.

**Figure 13:**
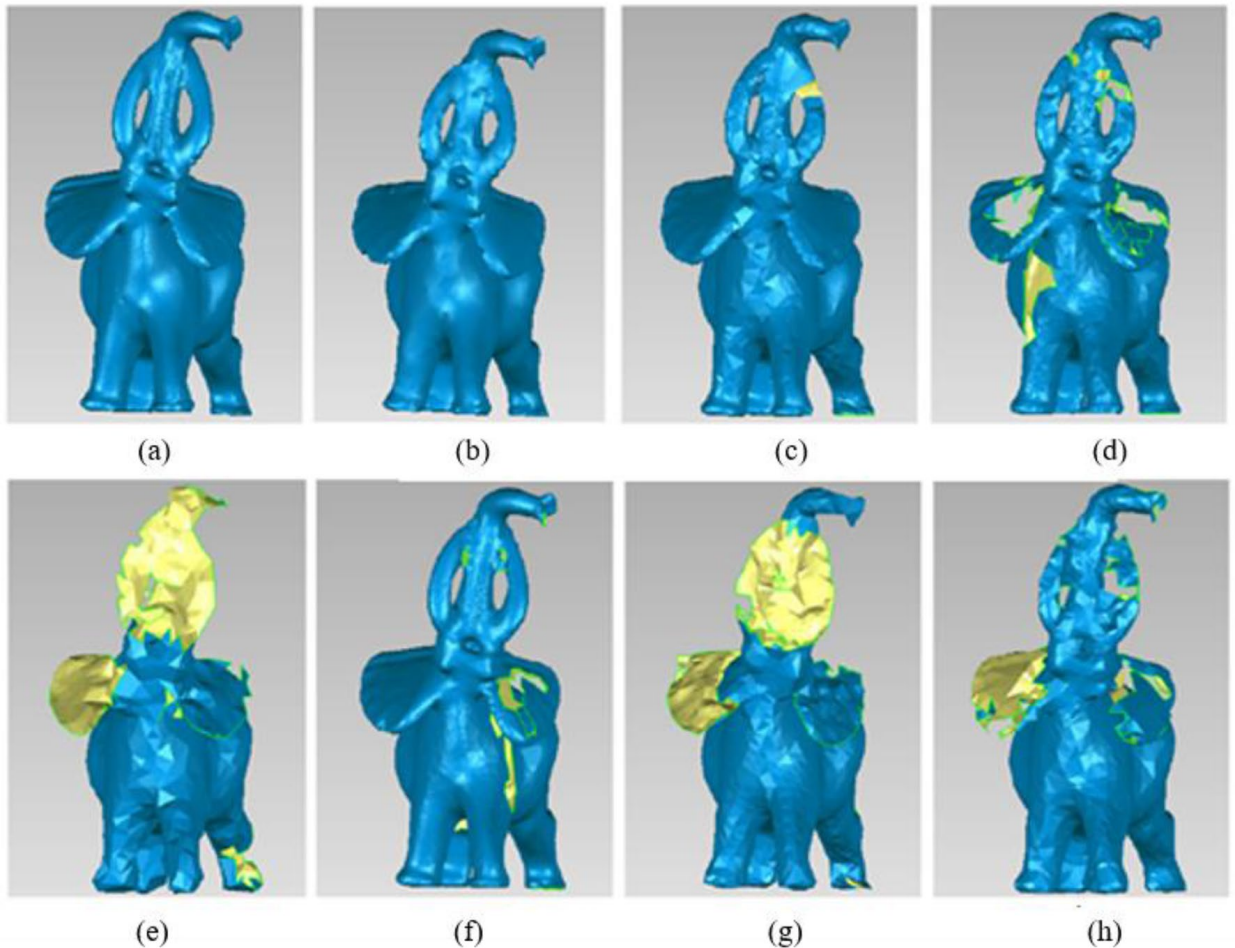
Different simplified results of Elephant. (**a**) Original data. (**b**) Our method. (**c**) The simplified method based on DFPSA. (**d**) The simplified method based on Gaussian spheres. (**e**) The simplified method based on octree coding. (**f**) The k-means clustering simplification method. (g) The uniform simplification method. (**h**) The geometric algebra method.

Figure 13 shows the reconstruction results of simplified elephant. (a) Original data, total number of the points = 24,955, (b) Our method, total number of the points = 7485, (c) The simplified method based on DFPSA, total number of the points = 8483, (d) The simplified method based on Gaussian spheres, total number of the points = 8591, (e) The simplified method based on octree coding, total number of the points = 2696, (f) The k-means clustering simplification method, total number of the points = 15,833, (g) The uniform simplification method, total number of the points = 2851, (h) The geometric algebra method, total number of the points = 3887.

Figure 13 illustrates that the simplification method based on octree coding and the uniform simplification method have seriously poor results in reconstruction. Not only are there large areas of holes, but also many other details lose. Except for the result of our method in (b), the other four methods have some small holes in nose, body or other parts. The reconstruction result generated by our method is nearly consistent with the original point cloud model and has fewer number of simplified points.

**Figure 14:**
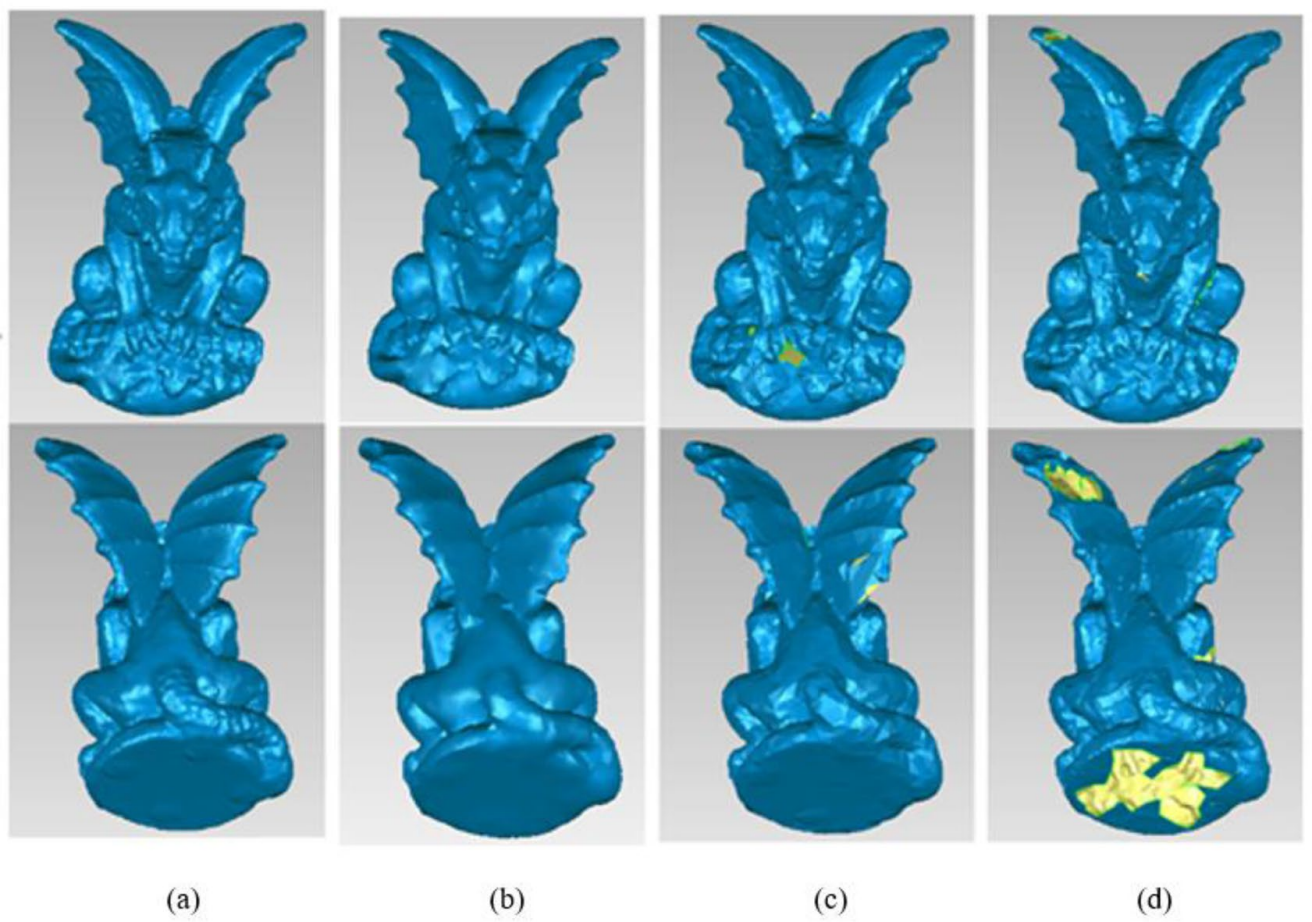
One of the reconstruction results of Gargo50k with different simplified methods. (**a**) Original data. (**b**) Our method. (**c**) The simplified method based on DFPSA. (**d**) The simplified method based on Gaussian spheres.

**Figure 15:**
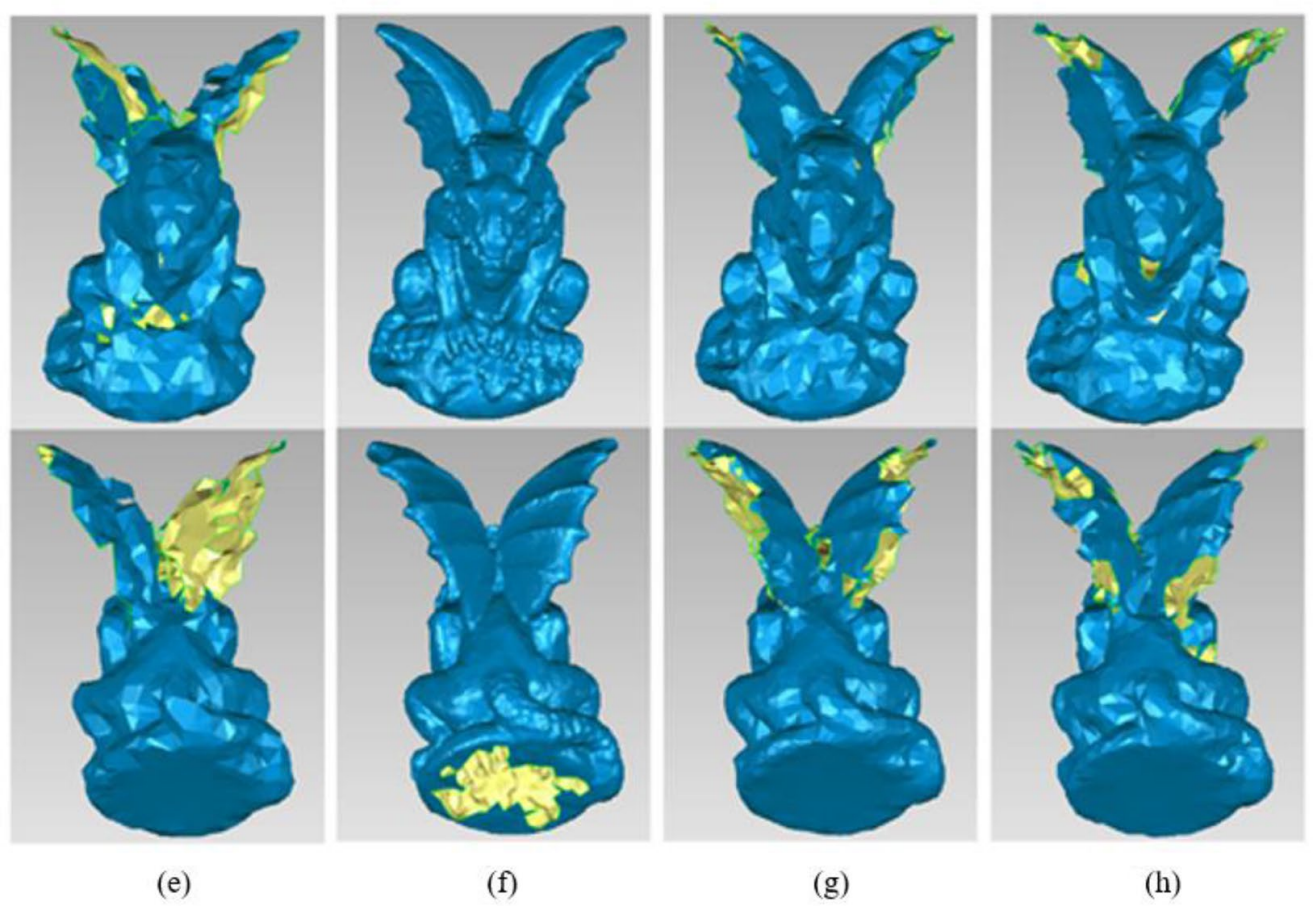
The other reconstruction results of Gargo50k with different simplified methods. (**e**) The simplified method based on octree coding. (**f**) The k-means clustering simplification method. (**g**) The uniform simplification method. (**h**) The geometric algebra method.

Due to the obvious asymmetry of the gargo50k, the front and the back side are both used to reconstruct 3D models for comparison explanation. The first row shows the front reconstruction results and the second row shows the back reconstruction results in Figs. 14 and 15.

The reconstruction results of gargo50k with different simplified methods are shown in Figs. 14 and 15. (a) Original data, total number of the points = 25,038, (b) Our method, total number of the points = 10,000, (c) The simplified method based on DFPSA, total number of the points = 12,604, (d) The simplified method based on Gaussian spheres, total number of the points = 16,138, (e) The simplified method based on octree coding, total number of the points = 2409, (f) The k-means clustering simplification method, total number of the points = 23,146, (g) The uniform simplification method, total number of the points = 3905, (h) The geometric algebra method, total number of the points = 3841.

The reconstruction results are not promising based on the octree coding and the uniform simplification method. There are large holes at the wing of gargo50k model. The simplified method based on k-means clustering has bad simplification rate, and the base at the back side still has holes. The methods based on the Gaussian spheres and geometric algebra also lead to a lot of holes, and there are vacancies on the front and back. Except for some small blanks, the DFPSA-based method has almost the same effect as our method proposed in this work. The overall analysis shows that the result generated by our method is superior to others.

**Figure 16:**
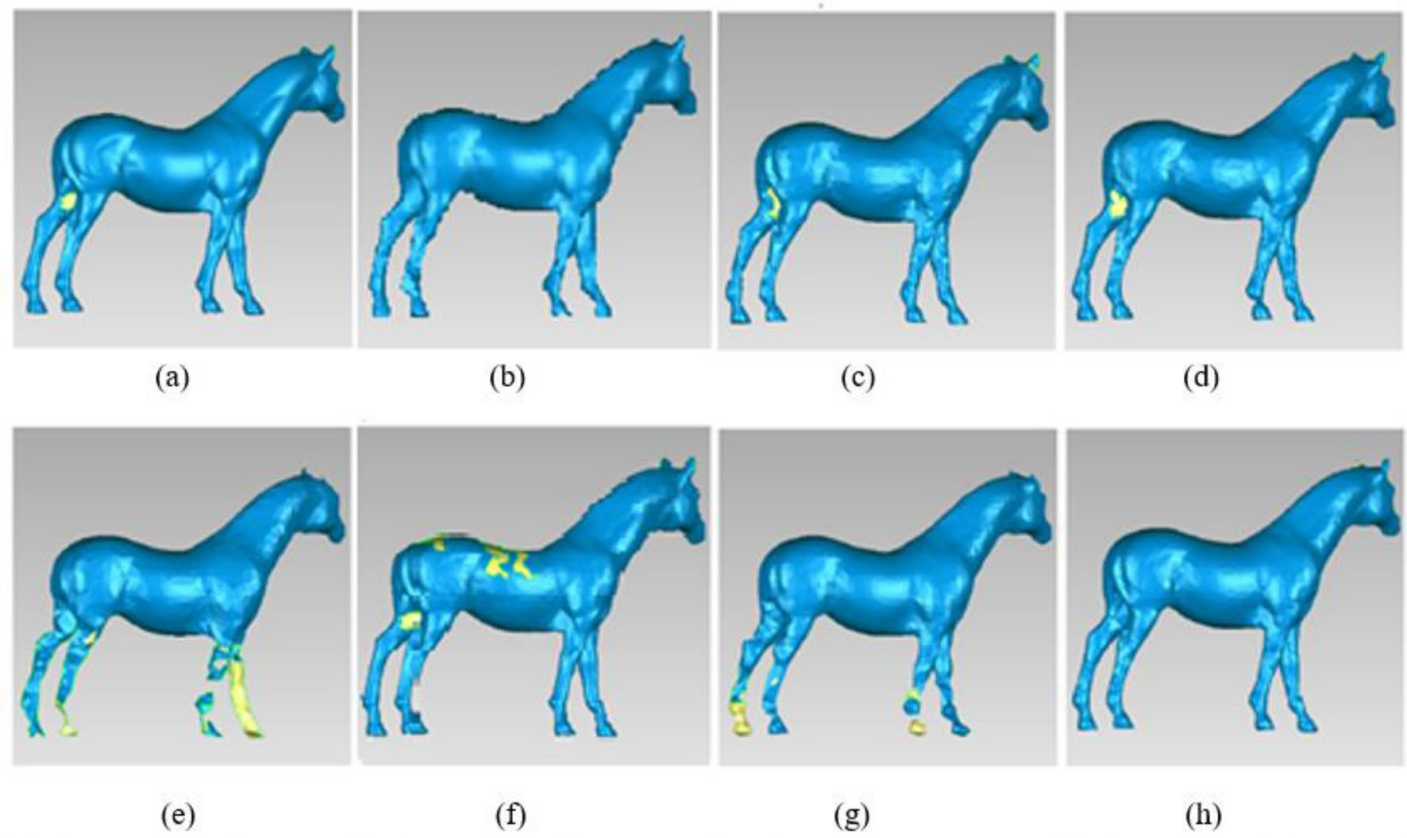
Different simplified results of Horse. (**a**) Original data. (**b**) Our method. (**c**) The simplified method based on DFPSA. (**d**) The simplified method based on Gaussian spheres. (**e**) The simplified method based on octree coding. (**f**) The k-means clustering simplification method. (**g**) The uniform simplification method. (**h**) The geometric algebra method.

Figure 16 shows the reconstruction results of horse. (a) Original data, total number of the points = 48,485, (b) Our method, total number of the points = 7000, (c) The simplified method based on DFPSA, total number of the points = 8107, (d) The simplified method based on Gaussian spheres, total number of the points = 10,058, (e) The simplified method based on octree coding, total number of the points = 2648, (f) The k-means clustering simplification method, total number of the points = 19,271, (g) The uniform simplification method, total number of the points = 4032, (h) The geometric algebra method, total number of the points = 7194.

In Fig. 16, the octree coding method and the uniform simplification method have high simplification rate, but many details of legs are lost, not only large holes, even faults. The method based on k-means clustering shows obvious details losing on the back of horse. The simplification results based on Gaussian spheres, DFPSA, and geometric algebra are almost the same as our method, and they are very close to the result of original data, but the total number of simplified point cloud by our method is fewer.

From the results of different simplification methods, we find that the simplification method based on octree coding and the uniform simplification method only retain the limited contours because of fewest points extracted, and there are holes in the model, and the simplification results are not good; the method based on geometric algebra has smaller number of simplified points, but in addition to the horse model, other models all show detail features losing. The k-means clustering method retains the detailed features to a large extent, but the simplification rate is lower, and some non-feature points also have holes, which makes the clustering method not ideal. Compared to the Gaussian sphere method and the DFPSA method, they maintain the surface feature contours, but our method has a higher simplification rate and has a better fitting effect according to the reconstruction results.

## Conclusion

Point cloud simplification plays a very important role in 3D data processing. One of the most important principles of point cloud simplification is to reduce the number of points as much as possible without affecting the reconstruction effect obviously. In this paper, a novel feature-preserving point cloud simplification framework is developed. It takes the advantages of deep learning in images and retains the geometric features and the potential surface of the point cloud, with higher reconstruction quality and fewer point numbers. The experimental results demonstrate that the proposed method is more universal to different models than other algorithms, and can better express the geometric appearance and detailed features of the 3D model. On the premise of the integrity of the model, our method can reach the highest simplification rate of the same point cloud. As for future work, the self-adaptive parameters should be developed. We hope this work can provide a useful data preprocessing tool for 3D model digitization.

## Methods

### Acquisition of the multi-angle 2D images

2D images are the projection of the 3D point cloud on a certain cross-section. To accurately describe the shape of the point cloud model with 2D images, the model needs to be rotated with multi-angles to obtain different images. Here, by writing a script file for the point cloud processing software (Geomagic Wrap), multi-angle 2D images are obtained by performing single axis variation of the model. It should be noted that the model is rotated around X-axis, Y-axis, and Z-axis respectively.

**Figure 17:**
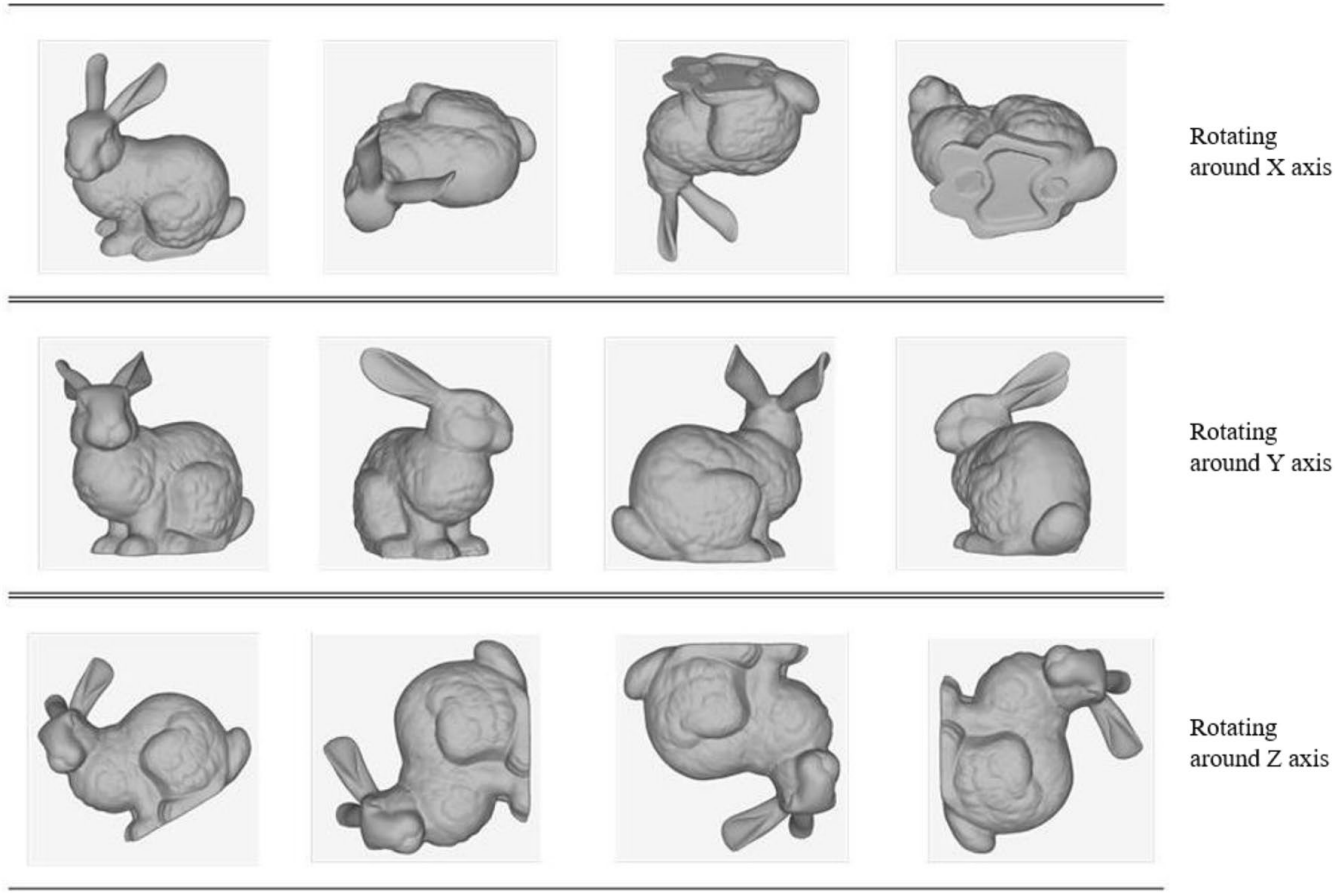
Schematic diagram of bunny model rotating around X/Y/Z axis.

As shown in Fig. 17, different axes provide different positions, and different rotation angles of the same axis also bring differences in feature points. The selecting of the rotating axis and the angle will be discussed later.

### Extraction of the surface feature lines

**Figure 18:**
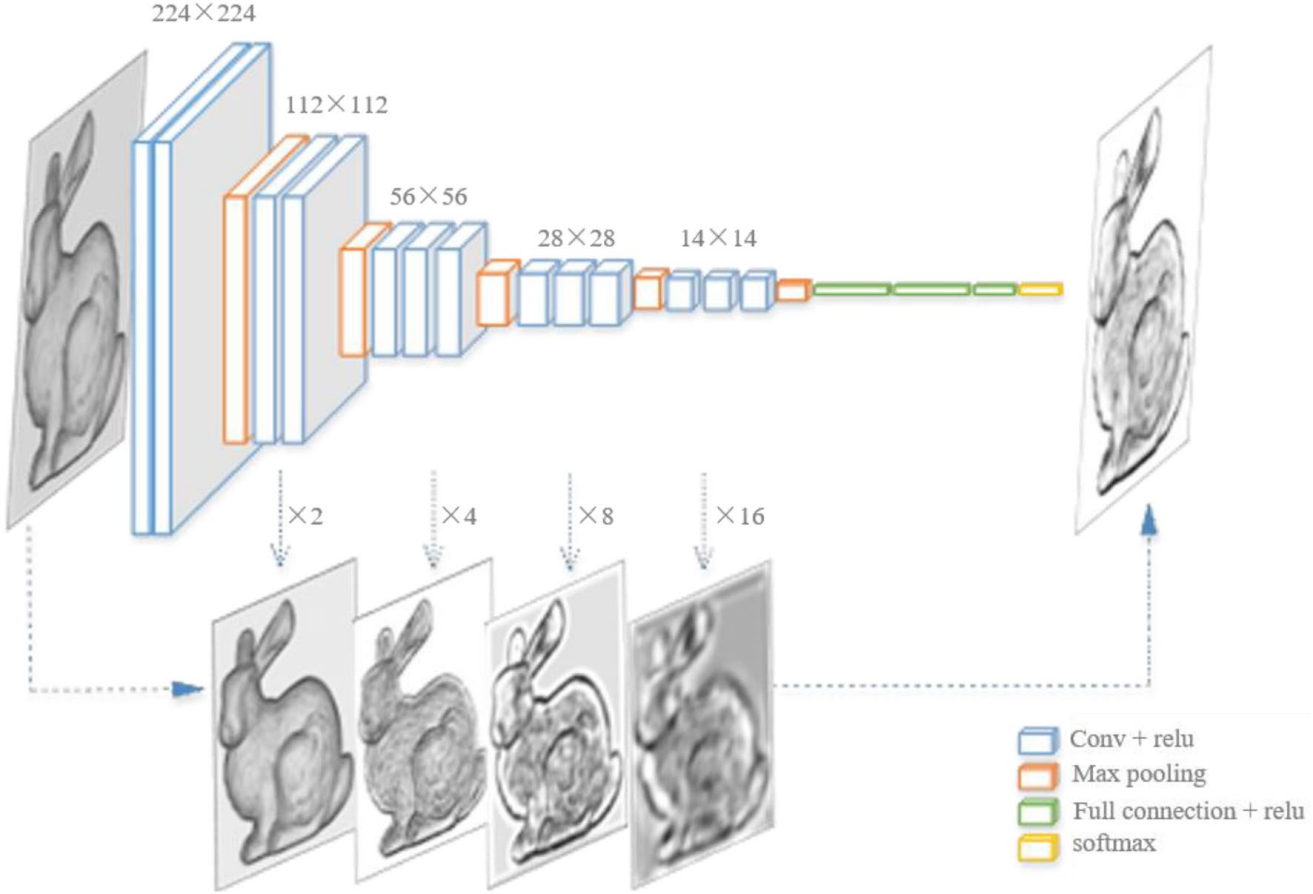
Feature line extraction neural network.

The neural network extracts feature lines contains five groups of convolutional layers, and the results of each side output layer are used for feature expression. An overview of the network is presented in Fig. 18. The network is mainly divided into two parts: one for feature extracting and the other for feature synthesis.

The feature extracting module is modified from the VGG-16^40^. The training dataset $S$ is $S = \left\{ {\left( {X_{n} ,Y_{n} } \right),n = 1, \ldots ,\left. N \right\}} \right.$,$X_{n} = \left\{ {X_{i} ,i = 1, \ldots ,\left. N \right\}} \right.$ represents the input images of the network, $Y_{n} = \left\{ {Y_{i} ,i = 1, \ldots ,\left. N \right\}} \right.$ represents the binary labels of $X_{n}$, $Y_{n} \in \left\{ 0 \right.,\left. 1 \right\}$, and $N$ refers to the number of input images. The dataset is fed into the network with 13 convolutional layers, 3 fully connected layers, and 5 pooling layers. The network has five stages of the side output for feature extracting and is deep supervised for each stage and the final fusion.

The feature synthesis module is to fuse feature maps of each stage. As shown in Fig. 18, the framework takes the image of Stanford’s bunny as input with the size of $224 \times 224$, and the size cuts in half every time when the image passes through each stage of convolutional layers, from $224 \times 224$ to $112 \times 112$, from $112 \times 112$ to $56 \times 56$, and drops until $14 \times 14$. The intermediate results are simultaneously extracted and saved with the size of $112 \times 112$, $56 \times 56$, $28 \times 28$, $14 \times 14$. These results are respectively enlarged at 2, 4, 8, and 16 times by deconvolution operation to make them consistent with the size of the input image. Finally, they are fused to output the grayscale feature image.

Each loss function used in this work is based on cross-entropy loss^41^ which can directly and clearly describe the relationship between the ground truth and prediction. The total loss is calculated by Eq. (1):

1 $$L_{total} = {\text{L}}_{stage} \left( {W,w} \right) + L_{fuse} \left( {W,h} \right),$$

$L_{total}$ describes the total loss of the network, ${\text{L}}_{stage} \left( {W,w} \right)$ is the sum of each branch loss, and $L_{fuse} \left( {W,h} \right)$ is the fusion loss of the mentioned branches. ${\text{W}}$ denotes the collection of all network layer parameters. $w = \left( {w^{\left( 1 \right)} , \ldots ,w^{\left( M \right)} } \right)$ denotes the weight of each stage, and $M$ is the number of stages, here is 5. ${\text{h}} = \left( {h_{1} , \ldots ,h_{M} } \right)$ is the fusion weight.

2 $${\text{L}}_{stage} \left( {W,w} \right) = \mathop \sum \limits_{k = 1}^{K} \alpha_{k} l_{stage}^{\left( k \right)} \left( {W,w^{\left( k \right)} } \right),$$

$\alpha_{k}$ in Eq. (2) is the weight parameter, and $l_{stage}^{\left( k \right)}$ denotes the loss function for the side output of stage $k$. The network is deep supervised and image-to-image training. All losses are trained equally and simultaneously. $L_{total}$ is minimized via standard (back-propagation) stochastic gradient descent to achieve the promising effect.

### Extraction of the feature points

According to the ideology of normalization, a mapping relationship is established between the feature lines of the 2D image and the feature points of 3D point cloud as follows.

3 $$x_{3}^{{\prime }} = \frac{{\max \left( {x_{3} } \right) - min\left( {x_{3} } \right)}}{m}*x_{2} + min\left( {x_{3} } \right)$$

4 $$y_{3}^{{\prime }} = \frac{{\max \left( {y_{3} } \right) - min\left( {y_{3} } \right)}}{n}*y_{2} + min\left( {y_{3} } \right)$$

$x_{3}$ and $y_{3}$ represent the values of X-axis and Y-axis coordinate of the 3D point cloud. $x_{3}^{{\prime }}$ and $y_{3}^{{\prime }}$ are the 3D coordinates of the candidate feature point. $x_{2}$ and $y_{2}$ represent the values of the X-axis and Y-axis coordinate of the 2D image. $\max \left( {x_{3} } \right)$ and $\max \left( {y_{3} } \right)$ refer to the maximum values of the X-axis and Y-axis coordinate of the 3D point cloud, $min\left( {x_{3} } \right)$ and $min\left( {y_{3} } \right)$ are the corresponding minimum values. $m$ and $n$ represent the length and width of the image. Based on mapping relationship in Eq. (3) and Eq. (4), the coordinates of X-axis and Y-axis for the candidate feature point can be roughly conformed. However, the coordinate of Z-axis cannot be determined directly. Here, we utilize $(x_{3}^{{\prime }} ,y_{3}^{{\prime }} )$ as the centroid to expand the filtering range of the surrounding point cloud. Therefore, the Eqs.3 and 4 can be updated to Eqs.5 and6. In this way, a point set P contained a series of candidate points is obtained from the point cloud.

5 $$x \in \left[ {x_{3}^{{\prime }} - \gamma \frac{{\max \left( {x_{3} } \right) - \min \left( {x_{3} } \right)}}{m},x_{3}^{{\prime }} + \gamma \frac{{\max \left( {x_{3} } \right) - min\left( {x_{3} } \right)}}{m}} \right]$$

6 $$y \in \left[ {y_{3}^{{\prime }} - \gamma \frac{{\max \left( {y_{3} } \right) - \min \left( {y_{3} } \right)}}{n},y_{3}^{{\prime }} + \gamma \frac{{\max \left( {y_{3} } \right) - \min \left( {y_{3} } \right)}}{n}} \right]$$

$\gamma$ is the expansion coefficient, which is used to control the number of candidate points. To characterize the point cloud as much as possible, the feature image obtained from the aforementioned network needs to be processed more finely. There are two main parameters $\alpha$ and $\beta$ to control the threshold of grayscale image and the boldness of the feature line. Three parameters $\alpha$, $\beta$ and $\gamma$ together control the quality of feature point acquisition.

In the point cloud, the same coordinates of X-axis and Y-axis often have multiple values of Z-axis. When selecting key points, all points in candidate point set P are calculated to obtain the average ${\overline{\text{z}}}$. As the 2D images are captured from the front side, that is, they are always at the positive direction of the Z-axis, and the corresponding Z-axis coordinate must be greater than the average ${\overline{\text{z}}}$. Based on this, we filter out the points with Z-axis coordinate less than the average ${\overline{\text{z}}}$ in point set P. In the remaining candidate point set P', the point with the smallest variance is the corresponding feature point. Finally, the whole feature points of the point cloud can be extracted.

### Non-feature point simplification

**Figure 19:**
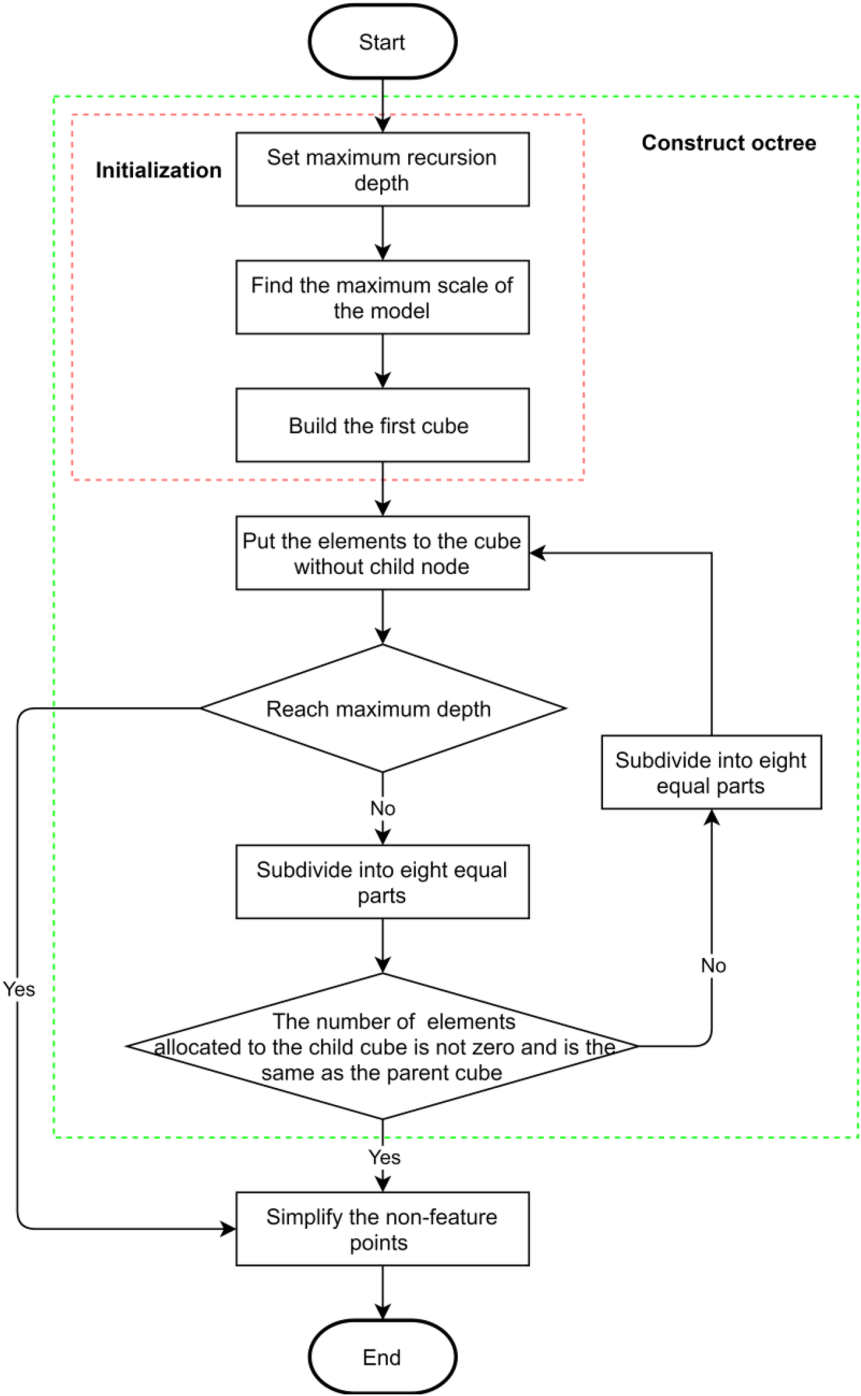
The flowchart of the non-feature point simplification.

As the non-feature points have a small amount of point cloud model information, they can be simplified to a great extent. The simplification method should be convenient and with low time complexity. For this purpose, an octree coding method is used for simplifying the non-feature points. The whole flowchart of the non-feature point simplification is shown in Fig. 19.

The octree coding construction consists of three steps. (1) Initialization: the maximum recursion depth, the maximum scale of the non-feature points, and the first cube. (2) The elements (here are points) are put into the cube without child node. (3) If the maximum depth is not reached, the octree coding subdivides the cube continuously, until the number of elements allocated to the child cube is not zero and is the same as the parent cube. The octree coding method is convenient and error-free when searching for voxels and corresponding points in voxels, and easy to balance the display accuracy and speed because of its orderly and layered characteristic.

As the non-feature points in the same leaf node are spatially close, we pay more attention to the relationship between the point and the whole leaf node. The average normal vector and the average curvature are used to describe the whole leaf node. In each octree leaf node, for all points located in it, the average normal vector $n_{avg}$ and the average curvature $c_{avg}$ of these points are calculated. Then, the difference between the normal vector $n$ of each point and $n_{avg}$ and the difference between the curvature $c$ of each point and $c_{avg}$ are calculated respectively. Moreover, the difference of normal vector and the curvature are added up. Finally, the point with the smallest sum value is selected to replace the other points in the leaf node.

Finally, the simplified point cloud can be obtained by fusing the feature points and non-feature points.

## Data Availability

The datasets analyzed during the current study available from the corresponding author on reasonable request.
